# Isolation and characterization of lytic phage TUN1 specific for *Klebsiella pneumoniae* K64 clinical isolates from Tunisia

**DOI:** 10.1186/s12866-021-02251-w

**Published:** 2021-06-21

**Authors:** Simone Eckstein, Jana Stender, Sonia Mzoughi, Kilian Vogele, Jana Kühn, Daniela Friese, Christina Bugert, Susann Handrick, Mustapha Ferjani, Roman Wölfel, Andrew Millard, Mohamed Ben Moussa, Joachim J. Bugert

**Affiliations:** 1 Bundeswehr Institute of Microbiology, Munich, Germany; 2 Department of Virology, Military Hospital of Instruction of Tunis, Tunis, Tunisia; 3 Faculty of Pharmacy, Monastir, Tunisia; 4 Department of Physics, Technical University of Munich, Garching, Germany; 5 Department of Anesthesiology and Reanimation, Military Hospital of Instruction of Tunis, Tunis, Tunisia; 6 Department of Genetics and Genome Biology, University of Leicester, Leicester, UK

**Keywords:** Multidrug-resistant bacteria, *Klebsiella pneumoniae*, Bacteriophage, phage therapy

## Abstract

**Background:**

Multidrug-resistant *Klebsiella pneumoniae spp. (kp)* are emerging agents of severe infections of the respiratory, urinary tract and wounds that can progress to fatal septicemia. The use of bacteriophages is currently being considered as an effective alternative or adjuvant to antibiotic therapy.

**Results:**

In this study, we report capsule (K)-typing of 163 carbapenem-resistant *Kp* (CRKP) isolated 2014–2018 at the Military Hospital of Instruction of Tunis (MHT), Tunisia, by partial amplification and sequencing of the *Kp wzi* gene. The most prevalent K-type overall was K64 with 50.3% followed by K17 and K27 (22.7 and 11.0%, respectively). K64 *Kp* strains were most common and associated with increased case/fatality rates, especially at the intensive care unit (ICU). Using a K64 *Kp* strain we isolated and characterized a lytic *Kp* phage, vB_KpP_TUN1 (phage TUN1), from wastewater samples of the ICU at the MHT. TUN1 belongs to the *Autographiviridae* family and specifically digests K64 *Kp* capsules most probably via a depolymerase encoded by *gp47*. Furthermore, we successfully assembled phage TUN1 in a non-replicative host (*E. coli*) raising the possibility of in vitro assembly in the absence of live bacterial hosts. We propose that phage TUN1 is a promising candidate to be used as an adjuvant or an alternative to antibiotic therapy in CRKP infections, facilitating regulatory approval of phage therapy.

**Conclusions:**

K64, K17 and K27 are the most common *wzi* capsule types in this geographical location in Northern Africa. The lytic phage TUN1 efficiently lyses K64 *Kp* strains associated with increased case/fatality rates at body temperature. Together with its ability to be rescued in a non-replicative host these features enhance the utility of this phage as an antibacterial agent.

**Supplementary Information:**

The online version contains supplementary material available at 10.1186/s12866-021-02251-w.

## Introduction

The spread of multidrug-resistant bacteria (MDRB) is an increasing global problem [1]. With reduced treatment options, the risk of life-threatening infections, including septicemia, grows and therefore new therapeutic approaches are required.

Bacteriophages targeting specific bacteria are considered a promising alternative to standard broad-spectrum antibiotic therapy, or an adjuvant to more specific antibiotic therapies, especially in the case of MDRB infections.

The ESKAPEE group of bacterial pathogens comprising *Enterococcus faecium*, *Staphylococcus aureus*, *Klebsiella pneumoniae*, *Acinetobacter baumannii*, *Pseudomonas aeruginosa*, *Enterobacter spp*., and *Escherichia coli* [2] highlights the clinical impact of MDRB, and illustrates the prevalence of multidrug-resistant gram-negative bacteria (MRGN) [3]. MRGN including *Klebsiella pneumoniae* (*Kp*) are also a problem in the military setting, indicated by the prevalence of MRGN in role 1 to 3 military treatment facilities [4]. MRGN can be resistant to up to 4 groups of antibiotics, which is why they are classified as MRGN1 to − 4, respectively. Among others, MRGN4 are resistant to carbapenem/ertapenem leaving colistin as the only remaining treatment option [5]. The emergence of carbapenem-resistant *Kp* strains (CRKP) and their spread in hospitals is an alarming phenomenon reported to be correlated with high case/fatality rate of 40–70% [6, 7]. Therefore, CRKP are important nosocomial pathogens involved in serious and life-threatening infections, especially in the intensive care unit (ICU) [8] and of particular concern to clinicians [9].

*Kp* cells have typical polysaccharides capsules, which form biofilms, confer resistance to antibiotics, reduce phagocytosis, prevent a vigorous immune response and hence are a major *Kp* virulence factor [10]. Currently, more than 80 *Kp* capsular types (K-types) are known [11]. The Wzi protein connects the polysaccharide capsule to the cell membrane of *Kp* [12]. In 2013, Brisse et al. developed a PCR-based assay correlating a 580 bp sequence of genomic *Kp wzi* with high statistical significance to K-types [13].

As mentioned above, there is an urgent need for therapeutic approaches for CRKP infections alternative to antibiotic treatment. Recently, the use of bacteriophages was shown to be effective in the treatment of MDRB in a landmark clinical study [14]. Bacteriophages specific for *Kp* have been isolated previously [15–18], including phage specific for capsule type K24 [19]. *Kp* phages contain K-type-specific enzymes that depolymerize the polysaccharides. Thereby the phage can reach the host surface and thus bind to a secondary receptor e.g. outer membrane proteins [20–22].

But, considering the wide variety of K-types, *Klebsiella* phages have to be isolated and characterized in large numbers to be used in therapeutic cocktails active against diverse *Kp* capsule types.

In this collaborative study between the Bundeswehr Institute of Microbiology (IMB) in Munich and the Military Hospital of Instruction of Tunis (MHT), we K-typed 168 CRKP obtained from a collection of strains, which were isolated from patients of different medical services at the MHT, Tunisia, between 2014 and 2018 [23] via *wzi* sequencing. Furthermore, we identified and characterized the lytic phage TUN1, a T7-type bacteriophage specific for *Kp* with the predominant capsule type K64 associated with increased case/fatality rates.

## Results

The majority of *Kp* strains analyzed in this study (70.2%) were isolated from patients in the MHT intensive care unit (ICU) followed by the unit for neonatology (10.7%). With < 3% each the remaining isolates were achieved from 13 other medical services at the MHT [23].

### The predominant capsule-type K64 is associated with increased case/fatality rates

To determine the K-type for each *Kp* strain, *wzi* gene sequencing was performed as described by Brisse et al. 2013 [13]. One hundred sixty-three of 168 *Kp* strains yielded the respective amplicon and 15 different K-types were found (Table 1). With 50.3% (82 CRKP strains), the most prevalent K-type was K64 followed by K17 and K27 (22.7 and 11.0%, respectively). For five *Kp* strains no amplification of the *wzi* gene locus could be achieved.

**Table 1:** K-genotype distribution of 163 clinical *Kp* strains

K-genotype	No. (%) of strains
K64	82 (50.3)
K17	37 (22.7)
K27	18 (11.0)
K15/K52	6 (3.7)
KN2	4 (2.5)
K25	4 (2.5)
K55	3 (1.8)
K24	2 (1.2)
K2	1 (0.6)
K3	1 (0.6)
K13	1 (0.6)
K43	1 (0.6)
K54	1 (0.6)
K62	1 (0.6)
KL105	1 (0.6)

**Fig. 1:**
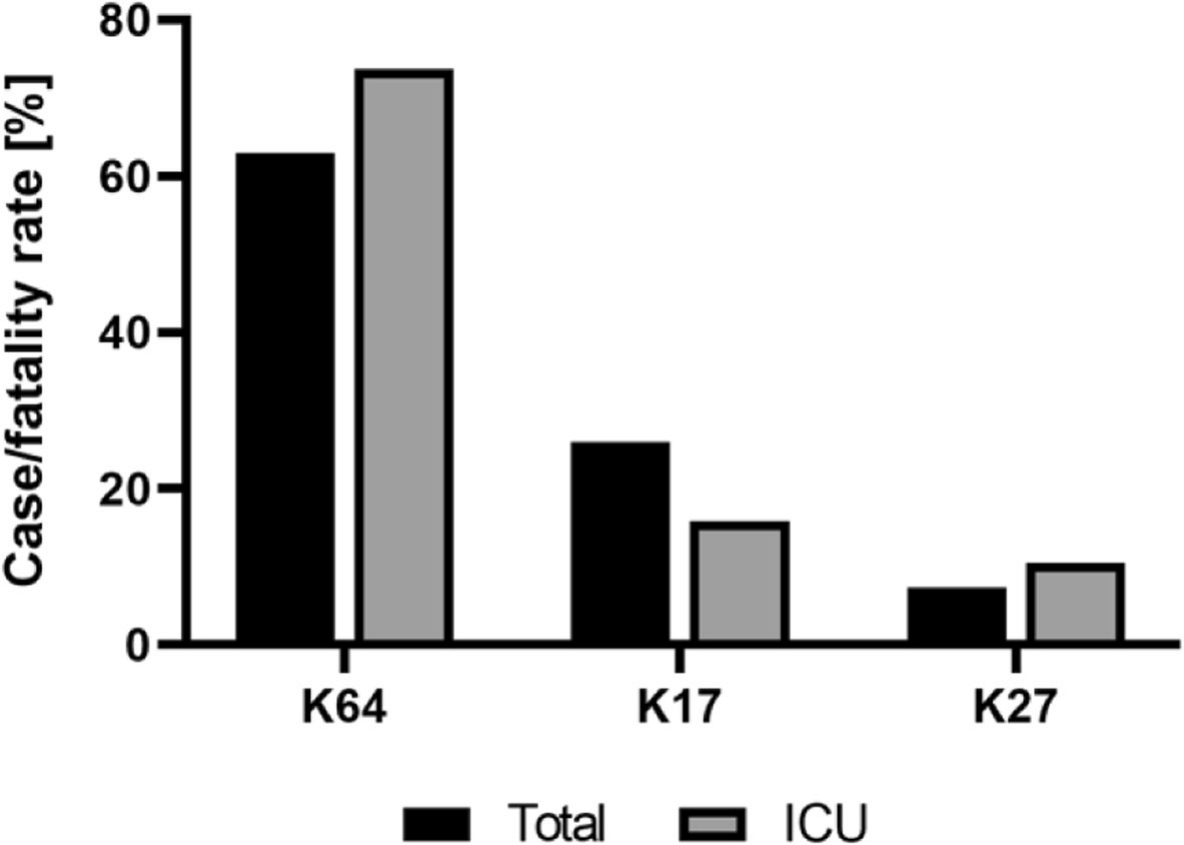
Case/fatality rates associated with *Kp* K-types K64, K17 and K27. Overall case/fatality rates (*black*) and case/fatality rates at the ICU (*grey*) in relation to CRKP-related deaths caused by *Kp* K64, K17 or K27 strains

With 27 CRKP-related deaths, the overall case/fatality rate of CRKP infections at the MHT lies at 18.0%. Looking at the pathogenicity of the three most common K-types, only K64 was associated with increased case/fatality especially at the ICU. With 63% in all units and 70.3% at the ICU almost two thirds of all CRKP-related deaths are therefore associated with *Kp* K64 infections (Fig. 1).

### *Kp* bacteriophage TUN1

**Fig. 2:**
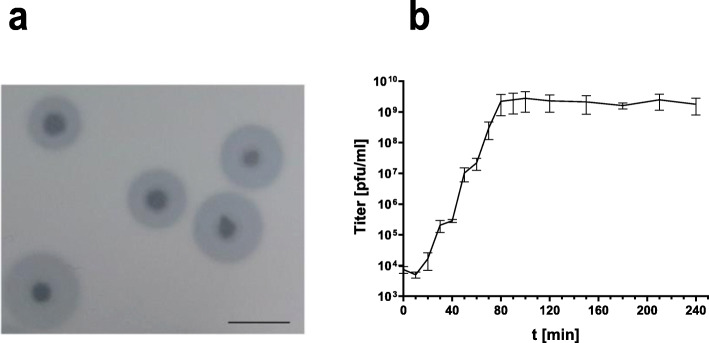
Initial characterization of phage TUN1 with its host *Kp* 7984. (**a**) Plaque assay revealing clear plaques each surrounded by a translucent halo. Scale bar: 50 mm (**b**) Growth curve of phage TUN1 was carried out at MOI = 1. Data is shown as mean from three biological experiments. Error bars represent standard deviation

To isolate bacteriophages specific for the Tunisian CRKP strains wastewater was collected from locations at the MHT. For phage enrichment the *Kp* host strain 7984, exhibiting the most predominant capsule type K64, identified in this study, was used. However, plaque-forming phages could only be found in the wastewater collected from the ICU. One of them, vB_KpP_TUN1 (phage TUN1), was further characterized in this study. Initial experiments showed that this phage forms central clear plaques (⌀ 1.5–2 mm) each surrounded by a halo (⌀ 5–6 mm after 12 h at 37 °C) on *Kp* 7984 (Fig. 2a). The growth curve showed that phage TUN1 has a replication cycle of 20 min and a burst size of 76 phage particles/cell (Fig. 2b).

### Phage TUN1 – a member of the *Autographiviridae* family – contains one putative depolymerase

**Fig. 3:**
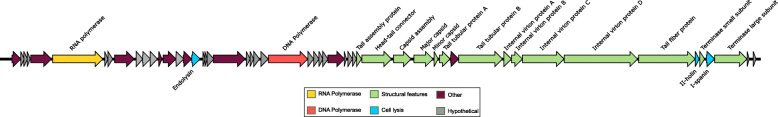
Schematic overview of the genome of phage TUN1. ORF coding for RNA polymerase is shown in *yellow*, while the ORF encoding a DNA polymerase is shown in *red*. ORFs coding for proteins involved in phage particle assembly and packaging are marked in *green*, and ORFs coding for enzymes involved in host lysis are marked in *blue*. Other functional ORFs are marked in *purple* while ORFs of hypothetical proteins are marked in *grey*. Arrows indicate the direction of transcription

Analyzing the genome of phage TUN1 using high-throughput sequencing revealed a linear genome with short direct terminal repeats consisting of 41,181 bp with a GC content of 52.9% and 53 predicted open reading frames (ORFs) as shown in Fig. 3 and Supplementary Table S1.

BlastN analysis found the most related genome sequence in the database to be *Klebsiella* phage 066037 (Accession No: MW042800.1, query coverage: 92 and 93.2% identity) belonging to the *Przondovirus* (*Kp32virus*) genus of the family *Autographiviridae* in the order of *Caudovirales* [24].

**Fig. 4:**
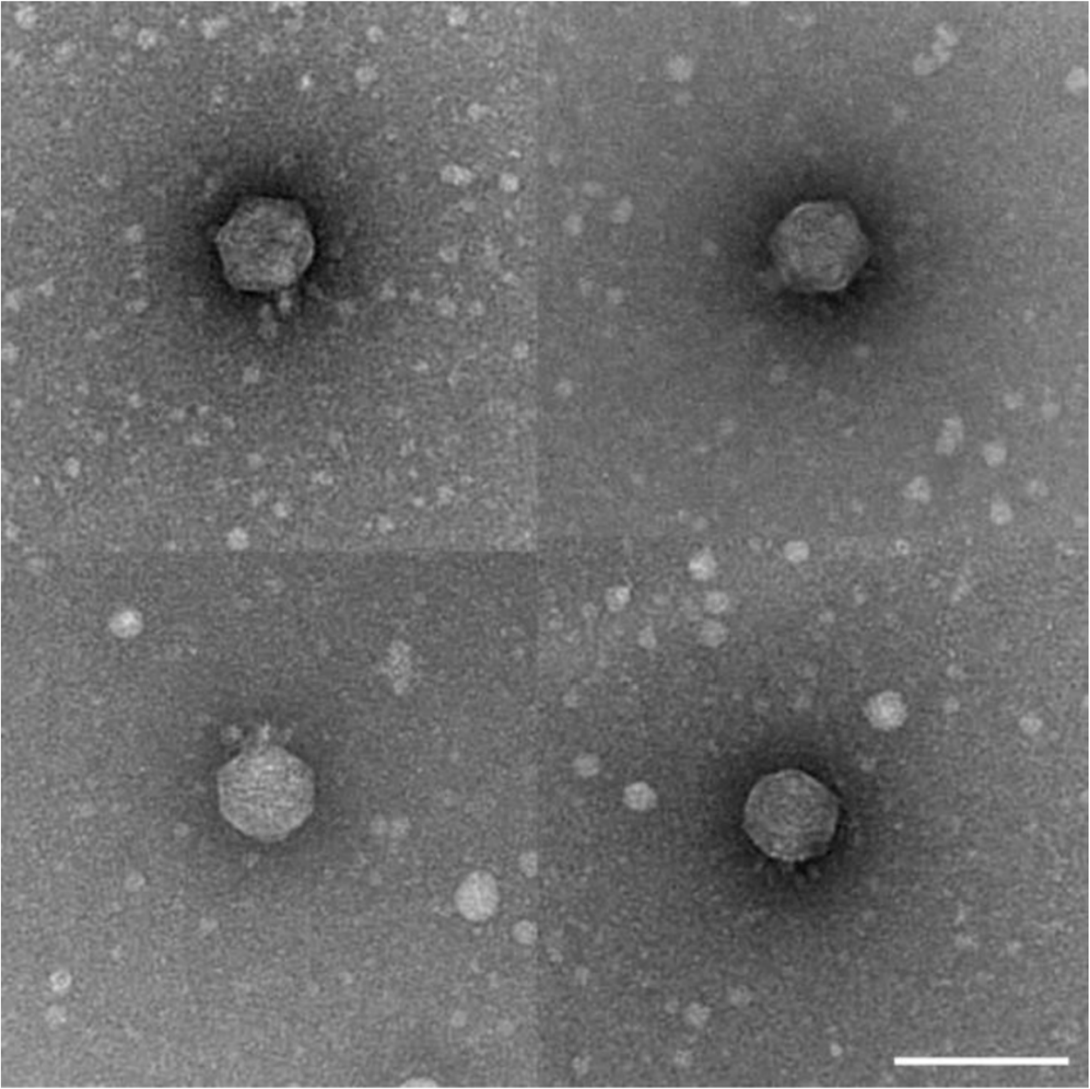
TEM of phage TUN1. Four TEM images showing one TUN1 phage particle each reveal the characteristic morphology of podoviruses*.* Scale bar: 100 nm

Examining the phage’s morphology using transmission electron microscopy (TEM), an icosahedral capsid with short tails could be observed (Fig. 4).

It has been described previously, that translucent halos surrounding phage plaques indicate the presence of phage-derived depolymerases [25]. Depolymerase activity of bacteriophages commonly lies within their tail fibers or tail spike proteins on the baseplate [26]. Nucleotide BLAST analysis revealed that the tail fiber protein, located downstream of internal virion protein D and upstream of class II holin (ORF 47 or *gp47*; Fig. 3, Supplementary Table S1), exhibits the highest similarity (query coverage: 97%; identity 96%) with the tail fibers protein of *Klebsiella* phage P509. This phage also exhibits specific depolymerase activity against K64 *Kp* strains [27]. Therefore, the tail fiber protein is most likely to exhibit depolymerase activity.

### Phage TUN1 shows a narrow host range restricted to K64 *Kp* strains

**Fig. 5:**
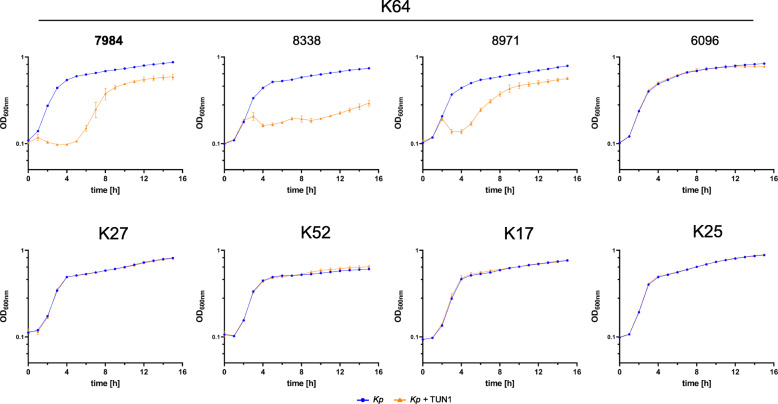
Lytic activity of phage TUN1 against *Kp* strains with different K-types. Upper panel: Growth experiments with phage TUN1 and four different Kp K64 strains. TUN1 lyses its host strain used for enrichment: *Kp* 7984 (written in bold) as well as *Kp* 8338 and 8971 with different efficiencies while *Kp* 6096 is not affected. Lower panel: Effect of phage TUN1 on *Kp* with the capsule types K27, K52, K17 and K25. Here, no lysis of *Kp* by phage TUN1 could be observed. *Green*: *Kp* strain alone. *Red*: *Kp* strain supplemented with phage TUN1

Since phage TUN1 was enriched on *Kp* 7984 a capsule type K64 strain, its specificity was investigated by testing 81 remaining Tunisian K64 *Kp* strains (Table 1) as well as representatives of other K-types as putative hosts. Eight representative strains are shown in Fig. 5. Phage TUN1 exhibits a narrow host range, as it was only able to lyse K64 *Kp* strains. Out of 82 K64 *Kp* strains 57 were lysed by phage TUN1, 25 were not, including strain 6096 shown as example in Fig. 5. None of the non-K64 capsule types were lysed.

### Rescue of phage TUN1 in non-pathogenic *E. coli* cells

For a potential therapeutic approach, genetic modification (e.g. to remove unwanted genetic elements like toxins, or genes allowing integration and a lysogenic state) and in vitro packing methods may be required, to facilitate regulatory approval. As a proof of principle that phage TUN1 can be rescued in a non-replicative host we transformed purified phage DNA into non-pathogenic *E. coli* NEBstable® 3040 cells. Although no plaque formation on *E. coli* could be observed (not shown), phage TUN1 assembly within the cells was successful, proven by subsequent plaque assays with *Kp* hosts. Here, 100 μl of a 10^− 2^ dilution of the supernatant of the transformation product containing TUN1 DNA resulted in 30 plaques. (Supplementary Figure S1).

## Discussion

The increasing spread of MRGN is a global problem [1]. Especially carbapenem-resistant *K. pneumoniae* (CRKP) pose a high risk in hospitals and other medical facilities. Liangfei Xu et al., reported 2-fold increased mortality among CRKP infected patients compared to those infected with carbapenem-susceptible *K. pneumoniae* (CSKP) [7].

Thus, alternative therapeutic approaches to antibiotic treatment are required. For CRKP infections, capsule-specific lytic bacteriophages are promising candidates [14, 17–19]. Knowledge of the K-type of the respective *K. pneumoniae* strains is therefore imperative.

This study reports the use of a rapid method to predict the K-type of *Kp* first described by Brisse et al. [13]. In total, 163 CRKP strains from a previously described Tunisian clinical specimen collection [Error! Reference source not found.] were capsule-typed. K-type K64 was not only identified as the most predominant K-type, but also as the K-type associated with the highest case/fatality rates of CRKP infected patients (> 70% in patients treated at the MHT ICU). This fits recent observations, both regarding the high case/fatality [6, 7] as well as the increased virulence of capsule type K64 [28].

However, the following limitations to this analysis have to be considered: i. the clinical specimen collection available lacks power in differentiating death caused by CRKP from any other factors, and it is difficult to draw definitive conclusions from current evidence because of the residual confounding factors and small sample sizes in many studies; ii. the study is retrospective in nature and thus susceptible to selection bias.

*Kp* can be found ubiquitously not only in human tissues but also in non-clinical sources, such as soil, drinking or surface waters and sewage [29]. This indicates that *Kp* phages should also be present in such environments and therefore easy to be isolated from nature.

In search of a potent tool to fight CRKP infections we looked for *Klebsiella* phages present at the MHT. Thereby we were able to obtain the lytic phage TUN1 from wastewater samples at the ICU using CRKP 7984 as a host strain. Based on BlastN analysis of the complete genome as well as morphologic characteristics phage TUN1 could be classified as bacteriophage of the genus *Przondovirus*, family *Autographiviridae* order *Caudovirales*, according to the newest species demarcation criteria set by the International Committee on Taxonomy of Viruses (ICTV) [23].

Phage TUN1 exhibited lysis specificity for Tunisian K64-type *Kp* strains. The replication cycle of 20 min and the burst size of 76 phage particles/cell are within the range described elsewhere [15–19]. The growth curve indicates that MOI = 1 reached approximately 60% of *Kp* cells, leading to a three-phased growth curve. While not classical, the three phases are distinguishable and similar enough to each other, to support the interpretation as a growth curve with 3 steps of 20 min, each, and a burst size of 76 particles per cell. As K64 was the most predominant K-type at the MHT, especially in the ICU, and all experiments were performed at 37 °C representing human body temperature, phage TUN1 appears to be a good candidate for phage therapy. Furthermore, unlike e.g. the mycobacterium abscessus phage used for therapy by Dedrick et al. [14] TUN1 is a purely lytic phage and therefore does not need removal of lysogenic elements form the genome.

However, with ~ 10 min, the latent time of phage TUN1 was found to be relatively short and the burst size with 76 phage particles/cell was on the lower end of productivity. Furthermore, the putative depolymerase seems to be very specific for K64 *Kp* strains.

Phage TUN1 was able to lyse more than two thirds but not all *Kp* strains of capsule type 64. Here, most probably factors other than capsule type like e.g. alternative receptor-binding proteins are involved in the process of phage-host recognition.

For future phage therapy applications, in addition to screening for naturally occurring broad-range bacteriophages, all desirable features such as strong lytic activity, absence of virulence factors and high phage stability could be combined via genetic engineering. The use of genetic elements with depolymerase activity, targeting additional clinically relevant capsule types, may lead to phages with broader host ranges, and therefore more promising candidates for phage therapy based on phage TUN1.

Conducting initial proof-of-principle experiments, we were able to show that phage TUN1 can be rescued in a non-replicative host (*E. coli*). In vitro assembly of therapeutic bacteriophages in the absence of live bacterial hosts would simplify the regulatory approval as the most common factors of concern like the presence of endotoxins or the possibility of host-DNA contamination, would be absent by default. A collaboration with the Walter Reed Army Institute of Research (WRAIR) is aiming for the development of technical approaches to field deployable therapeutic phage cocktails [30, 31].

The future goals of our current project at the Bundeswehr Institute of Microbiology are to i. identify and characterize bacteriophages against a broad range of *Kp* K-types found in German/Tunisian military and civilian hospitals; ii. develop technical approaches that allow genetic modification for the removal of unwanted genetic/lysogenic phage genome elements, possibly the addition of capsule specific factors and iii. Work out a pipeline for therapeutic phage production, that implements the highest safety standards, facilitating regulatory approval.

Such a pipeline could then be applied in both civilian and military hospitals, and eventually extended to products able to withstand the harsher conditions in military field settings.

## Conclusion

In conclusion, 163 carbapenem-resistant *K. pneumonia* strains from a previously published MHT collection were capsule-typed. Furthermore, a lytic phage, vB_KpP_TUN1, specific for *Kp* capsule type K64 was isolated from wastewater samples and analyzed for its host range, morphology and genomic characteristics. K64, K17 and K27 were the most common *wzi* capsule types in this geographical location in Northern Africa, at the MHT, with K64 being predominant overall and in the ICU, were it caused increased case fatality rates. Geographical prevalence of K-types is a rationale behind priority phage production for therapy. Therefore, compassionate use of GMP-produced K64 *Kp*-specific bacteriophages at the MHT to control infections caused by multidrug-resistant *K. pneumonia* as adjuvant to antibiotic therapy is considered to be the next step forward. The genome of vB_KpP_TUN1 is devoid of lysogenic elements or toxins and therefore safe for use according to current standards in phage therapy. Future studies will focus on further improving the effectiveness in terms of host range and safety, of *Kp*-specific bacteriophages, for example by identification and removal of non-essential genes.

## Materials and methods

### Clinical Kp strains used in this study

One hundred sixty-eight clinical *Kp* strains were isolated and characterized as described elsewhere following the operating procedure of the Military Hospital of Tunis [23]. Isolates were recovered from anal swabs (*n* = 32), axillary swabs (*n* = 2), a swab from a not-further defined location (*n* = 1), materials from broncho-alveolar lavages (BAL) (*n* = 5), blood cultures (*n* = 43), cytobacteriological examinations of sputum (CBES) (*n* = 5), cerebrospinal fluid (CSF) (*n* = 1), exudates from the ear (*n* = 2), biofilms at endotracheal tubes (*n* = 2), gastric samples (*n* = 1), catheters (*n* = 19), oral swabs (*n* = 1), protected tracheal sampling materials (PTS) (*n* = 12), PUS (*n* = 15), a medical device (*n* = 1), and urine (*n* = 26). A list of the different sample sources linked to the 163 identified capsule types can be found in Supplementary Table S2.

### Kp capsule typing

To determine *Kp* capsular types (K-types), PCR-amplification of a 580-bp fragment of the *wzi* gene was performed according to Brisse et al., 2013 [13]. The respective PCR product was gel purified (QIAquick Gel Extraction Ki, Qiagen, Germany) and subsequently sent for sequencing (Eurofins Genomics, Germany).

*wzi* sequences were analyzed against the NCBI Genbank using BLASTN and the K-type alignments with the highest scores selected.

### Bacteriophage isolation

Bacteriophages were isolated from wastewater samples from different locations in the MHT. To select bacteriophages, 10 mL of the sample was clarified by centrifugation at 7000 x g for 10 min at 4 °C, followed by filtration of the supernatant through a 0.2 μm pore size filter. 6.5 ml of the water sample was then mixed with 6 ml LB + 1 mM CaCl_2_ solution and 60 μl of host bacterium using the strain 7984 (a K64-type *Kp*, the most common type found in the Tunis military hospital). For the host an over night (O/N) culture of the chosen host strain was diluted 1:100 in LB medium and grown until it reached an OD_600_ of 0.4–0.5.

Subsequently, the mixture was incubated at 37 °C for 42 h. After 42 h the bacteria phage mixture was centrifuged at 7000 x g for 10 min at 4 °C to spin down most bacteria. The supernatant was then filtrated through a 0.2 μm pore size filter using a 20 ml syringe into a sterile tube. The filtrate, containing possible phages, was stored at 4 °C until further use.

### Plaque assay and plaque purification

Plaque assays were conducted as described elsewhere [31]. In brief, 100 μl filtrated phage-solution were mixed with 350 μl of host *Kp* (OD_600_nm = 0.4–0.6). 2.5 ml of 0.6% LB-agar (40 °C) containing 1 mM CaCl_2_ were added and poured on a pre-prepared 2% (w/v) LB-agar plate. When the top-layer has hardened the plates were inverted and incubated O/N at 37 °C.

To purify a phage plaque. *Kp* strains were grown in LB-broth containing 1 mM CaCl_2_ (37 °C, shaking) to OD_600_ = 0.4–0.6. A single plaque from a previous plaque assay (based on size, reachability, representativeness) was picked with a sterile pipette tip, resuspended in a 15 mL Falcon with 350 μl bacteria and filled up with LB-broth to 5 ml. The tube was incubated (37 °C, shaking) for 3–6 h. To remove the remaining bacteria the tube was centrifuged at > 7000 g, for 10 min, ate 4 °C. The supernatant was filtrated through a 0.2 μm pore size filter under sterile conditions. The stock was stored at 4 °C until further use.

### Tem

The purified phage solution was added to glow-discharged formvar-supported carbon-coated Cu400 TEM grids (FCF400-CU, Science Services, Munich, Germany) for 30 s, followed by a negative stain using a 2% aqueous uranyl formate solution with 25 mM sodium hydroxide for 45 s. This step is followed by a drying step under vacuum for 30 min. For imaging a Philips CM100 transmission electron microscope at 100 kV was used. For acquiring images an AMT 4 megapixel CCD camera was used and was performed at a magnification between × 15,500 and × 21,000. For image processing the plugin Scale Bar Tools for Microscopes for Java-based software ImageJ was used (version 1.80).

### Host-range determination

Single colonies of *Kp* strains from a LB-agar-plate were resuspended in 5 mL LB-broth and 100 μl each were transferred into wells of a 96-well plate.

The phage stock was diluted to 10^4^ pfu/ml and 100 μl of phage or LB-broth were added to the bacteria. Bacterial growth was then monitored by measuring the OD_600nm_ every 60 min for 16 h at 37 °C under static conditions using a TECAN Spark Multireader.

### TUN1 growth curve

Growth experiments were done as described by Delbrück and Ellis in 1939 [32]. Briefly, all *Kp* strains were grown in LB-broth containing 1 mM CaCl_2_ (37 °C, shaking) to OD_600_nm = 0.4–0.6. *Klebsiella* cells were counted in a haemocytometer and an infection with a MOI = 1 was prepared using a concentration of 1 × 10^4^ in 10 mL LB-broth.

Every 10–30 min a plaque assay with different dilutions (10^0^–10^− 2^) using the top-layer method with LB-agar (0.6%, 40 °C), containing 1 mM CaCl_2_, was performed. The plates were inverted and incubated overnight. On the next day titer and burst size were calculated by counting the plaque forming units (PFUs) using the formula: median number of plaques of t_end of the replication cycle_/t_start of the replication cycle_ for MOI = 1.

### Genome analysis

The phage DNA was prepared with Lucigen MasterPure™ Complete DNA and RNA Purification Kit (Cat.No. MC85200). Approximately 1 μg of DNA was used in library preparation for minION sequencing using the ligation sequencing kit (SQK-LSK109) following the manufacturer’s instructions (Oxford Nanopore Technology, UK). Base-calling was carried out with guppy v3.4.3. Genome assembly was carried out with Unicycler v0.4.5 [33] using all reads > 5 kb in length, with further polishing using Medaka. The genome was initially annotated with Prokka, with further annotation using the RAST server [34], followed by manual curation. Genomic termini were established using PhageTerm [35]. As the inbuilt aligner of PhageTerm does not map long reads a work around was developed. Reads were with mininmap2 v2.1 with the map-ont option and the start position and strand of each read extracted from the PAF file. This information was used to create pseudo set of short reads, by truncation of all reads to 150 nucleotides in length whilst maintaining the start position extracted from the long read data, required to determine the termini. This data was used input into PhageTerm using the –f option.

Sequencing was conducted from DNA samples by the MWG Eurofins GATC sequencing service.

### Phage DNA transformation

Five μl of purified phage DNA were added to *E. coli* NEBstable C3040 and transformed by heatshock (42 °C for 90 s). Subsequently, 1 ml LB-broth was added and the bacteria were grown at 37 °C, shaking. After 2 h, 500 μl of the transformed bacteria solution were centrifuged and the supernatant was kept at 4 °C to perform subsequent plaque and spot assays on *Kp* strains.

## Supplementary Information

**Additional file 1: Supplementary Table S1.** Gene localizations (nucleotides), lengths (bp) and products of predicted phage TUN1 ORFs. **Supplementary Table S2.** Correlation of *Kp* capsule types and sample origin. List of the different capsule types for the 163 *Klebsiella pneumoniae* strains analyzed in this study sorted by sample origin. Capsule types were predicted by partial *wzi* sequencing. **Supplementary Figure S1.** Plaque assay of *Kp* 7984 with phage TUN1 assembled in *E. coli* NEBstable cells.

## Data Availability

The phage genome datasets generated and analysed during the current study are available at the European Nucleotide Archive (ENA) - https://www.ebi.ac.uk/ena/browser/view/PRJEB37291; sequence ID ERZ1741895. Furthermore, the assembly is also available at NCBI under the accession number HG994092.1 (https://www.ncbi.nlm.nih.gov/nuccore/HG994092.1).

## Abbreviations

CRKP: Carapenem-resistant *Klebsiella pneumoniae*
CSKP: Carbapenem-susceptible *Klebiella pneumoniae*
ICU: Intensive care unit
IMB: Bundeswehr Institute of Microbiology
K-type: Capsule type
K64: Capsule type 64
*Kp*: *Klebsiella pneumoniae*
MDRB: Multidrug-resistant bacteria
MHT: Military Hospital of Instruction of Tunis
MOI: Multiplicity of infection
MRGN: Multidrug-resistant gram-negative bacteria
ORF: Open reading frame
TEM: Transmission electron microscopy
