# Supplementary material for: Isolation and characterization of lytic phage TUN1 specific for Klebsiella pneumoniae K64 clinical isolates from Tunisia
Source: BMC Microbiol. 2021 Jun 21;21:186. doi: 10.1186/s12866-021-02251-w (PMC8215767; doi:10.1186/s12866-021-02251-w)
Supplement: Supplementary file 1 — Additional file 1: Supplementary Table S1. Gene localizations (nucleotides), lengths (bp) and products of predicted phage TUN1 ORFs. Supplementary Table S2. Correlation of Kp capsule types and sample origin. List of the different capsule types for the 163 Klebsiella pneumoniae strains analyzed in this study sorted by sample origin. Capsule types were predicted by partial wzi sequencing. Supplementary Figure S1. Plaque assay of Kp 7984 with phage TUN1 assembled in E. coli NEBstable cells. [file 12866_2021_2251_MOESM1_ESM.pdf]

Supplementary material for manuscript:

Isolation and characterization of lytic phage TUN1 specific for *Klebsiella pneumoniae* K64 clinical isolates from Tunisia

Simone Eckstein [1,†], Jana Stender [1,†], Sonia Mzoughi [2,3,†], Kilian Vogele [4], Jana Kühn [1], Daniela Friese [1], Christina Bugert [1], Susann Handrick [1], Mustapha Ferjani [5], Roman Wölfel [1], Andrew Millard [6], Mohamed Ben Moussa [2], and Joachim J Bugert [1,*]

[1] Bundeswehr Institute of Microbiology, Munich, Germany
[2] Department of Virology, Military Hospital of Instruction of Tunis, Tunisia
[3] Faculty of Pharmacy, Monastir, Tunisia
[4] Department of Physics, Technical University of Munich, Garching, Germany
[5] Department of Anesthesiology and Reanimation, Military Hospital of Instruction of Tunis, Tunisia
[6] Department of Genetics and genome Biology, University of Leicester, UK

† These authors contributed equally
* Correspondence: Joachim1Bugert@bundeswehr.org

Content:

- **Supplementary Table S1.** Gene localizations (nucleotides), lengths (bp) and products of predicted phage TUN1 ORFs.

- **Supplementary Table S2. Correlation of *Kp* capsule types and sample origin.** List of the different capsule types for the 163 *Klebsiella pneumoniae* strains analyzed in this study sorted by sample origin. Capsule types were predicted by partial *wzi* sequencing.

- **Supplementary Figure S1. Plaque assay of *Kp* 7984 with phage TUN1 assembled in *E. coli* NEBstable cells**.

Supplementary Table S1. Gene localizations (nucleotides), lengths (bp) and products of predicted phage TUN1 ORFs.

ORF	Gene product	Start	End	Length	Direction
ORF_1	S-adenosyl-L-methionine hydrolase	658	1,125	468	forward
ORF_2	hypothetical protein	1,125	1,313	189	forward
ORF_3	hypothetical protein	1,291	1,488	198	forward
ORF_4	hypothetical protein	1,472	1,642	171	forward
ORF_5	kinase	1,674	2,780	1,107	forward
ORF_6	RNA polymerase	2,850	5,570	2,721	forward
ORF_7	hypothetical protein	5,670	5,846	177	forward
ORF_8	hypothetical protein	5,850	6,107	258	forward
ORF_9	ATP-dependent DNA ligase	6,208	7,266	1,059	forward
ORF_10	hypothetical protein	7,376	7,726	351	forward
ORF_11	hypothetical protein	7,719	8,147	429	forward
ORF_12	HNH Endonuclease	8,134	8,523	390	forward
ORF_13	inhibitor of host bacterial RNA polymerase	8,600	8,764	165	forward
ORF_14	ssDNA-binding protein	8,831	9,529	699	forward
ORF_15	hypothetical protein	9,529	9,948	420	forward
ORF_16	endonuclease I	9,929	10,375	447	forward
ORF_17	endolysin (lysozyme/N-acetylmuramoyl-L-alanine amidase)	10,378	10,833	456	forward
ORF_18	hypothetical protein	10,983	11,099	117	forward
ORF_19	hypothetical protein	11,096	11,215	120	forward
ORF_20	HNH Endonuclease	11,212	11,577	366	forward
ORF_21	primase/helicase protein	11,552	13,270	1,719	forward
ORF_22	hypothetical protein	13,335	13,544	210	forward
ORF_23	hypothetical protein	13,545	13,727	183	forward
ORF_24	hypothetical protein	13,740	14,051	312	forward
ORF_25	hypothetical protein	14,124	14,525	402	forward
ORF_26	DNA polymerase	14,542	16,659	2,118	forward
ORF_27	hypothetical protein	16,674	16,979	306	forward
ORF_28	hypothetical protein	16,983	17,273	291	forward
ORF_29	hypothetical protein	17,270	17,479	210	forward
ORF_30	hypothetical protein	17,476	17,760	285	forward
ORF_31	exonuclease	17,753	18,658	906	forward
ORF_32	hypothetical protein	18,640	18,750	111	forward
ORF_33	hypothetical protein	18,836	19,081	246	forward
ORF_34	hypothetical protein	19,084	19,305	222	forward
ORF_35	tail assembly protein	19,307	19,567	261	forward
ORF_36	head-tail connector protein	19,591	21,198	1,608	forward
ORF_37	capsid assembly protein	21,302	22,255	954	forward

ORF_38	major capsid protein	22,410	23,441	1,032	forward
ORF_39	predicted minor capsid protein	23,498	23,719	222	forward
ORF_40	tail tubular protein A	23,788	24,366	579	forward
ORF_41	endonuclease VII	24,392	24,832	441	forward
ORF_42	tail tubular protein B	24,829	27,204	2,376	forward
ORF_43	internal virion protein A	27,277	27,687	411	forward
ORF_44	internal virion protein B	27,690	28,280	591	forward
ORF_45	internal virion protein C	28,280	30,535	2,256	forward
ORF_46	internal virion protein D	30,552	34,517	3,966	forward
ORF_47	tail fiber protein	34,579	37,632	3,054	forward
ORF_48	class II holin	37,643	37,852	210	forward
ORF_49	terminase small subunit	37,886	38,143	258	forward
ORF_50	i-spanin	38,240	38,689	450	forward
ORF_51	terminase large subunit	38,682	40,439	1,758	forward
ORF_52	hypothetical protein	40,449	40,556	108	forward
ORF_53	hypothetical protein	40,780	40,929	150	forward

Supplementary Table S2. Correlation of Kp capsule types and sample origin. List of the different capsule types for the 163 *Klebsiella pneumoniae* strains analyzed in this study sorted by sample origin. Capsule types were predicted by partial *wzi* sequencing.

Sample origin	Capsule type (Total No. / percentage [%])
Anal swabs	**32 / 19.6**
	K13 (1 / 3.1)
	K17 (3 / 9.4)
	K25 (2 / 6.3)
	K27 (4 / 12.5)
	K64 (22 / 68.8)
Axillary sampling	**2 / 1.2**
	K27 (2 / 100)
BAL	**5 / 3.1**
	K17 (1 / 20.0)
	K27 (1 / 20.0)
	K52 (1 / 20.0)
	K64 (2 / 40.0)
Blood culture	**41 / 25.2**
	K17 (11 / 26.8)
	K27 (2 / 4.9)
	K43 (1 / 2.4)
	K52 (1 / 2.4)
	K55 (2 / 4.9)
	K62 (1 / 2.4)

	K64 (22 / 53.7)
	KN2 (1 / 2.4)
CBES	**5 / 3.1**
	K17 (1 / 20.0)
	K27 (1 / 20.0)
	K64 (2 / 40.0)
	KN2 (1 / 20.0)
CSF	**1 / 0.6**
	K64 (1 / 100)
Ear exudates	**2 / 1.2**
	K25 (1 / 50.0)
	K64 (1 / 50.0)
Endotracheal tube	**2 / 1.2**
	K17 (2 / 100)
Gastric levy	**1 / 0.6**
	K17 (1 / 100)
Catheter	**18 / 11.0**
	K3 (1 / 5.6)
	K17 (2 / 11.1)
	K27 (2 / 11.1)
	K52 (2 / 11.1)
	K64 (11 / 61.1)
Oral swab	**1 / 0.6**
	K17 (1 / 100)
PTS	**10 / 6.1**
	K2 (1 / 10.0)
	K17 (1 / 10.0)
	K27 (2 / 20.0)
	K55 (1 / 10.0)
	K64 (5 / 50.0)
PUS	**15 / 9.2**
	K17 (6 / 40.0)
	K27 (2 / 13.3)
	K52 (1 / 6.7)
	K64 (5 / 33.3)
	KL 105 (1 / 6.7)
Medical device	**1 / 0.6**
	K64 (1 / 100)
Urine	**26 / 16.0**
	K17 (9 / 34.6)

	K24 (1 / 3.8)
	K25 (1 / 3.8)
	K27 (1 / 3.8)
	K52 (1 / 3.8)
	K54 (1 / 3.8)
	K55 (1 / 3.8)
	K64 (9 / 34.6)
	KN2 (2 / 7.7)
Undefined	**1 / 0.6**
	K64 (1 / 100)

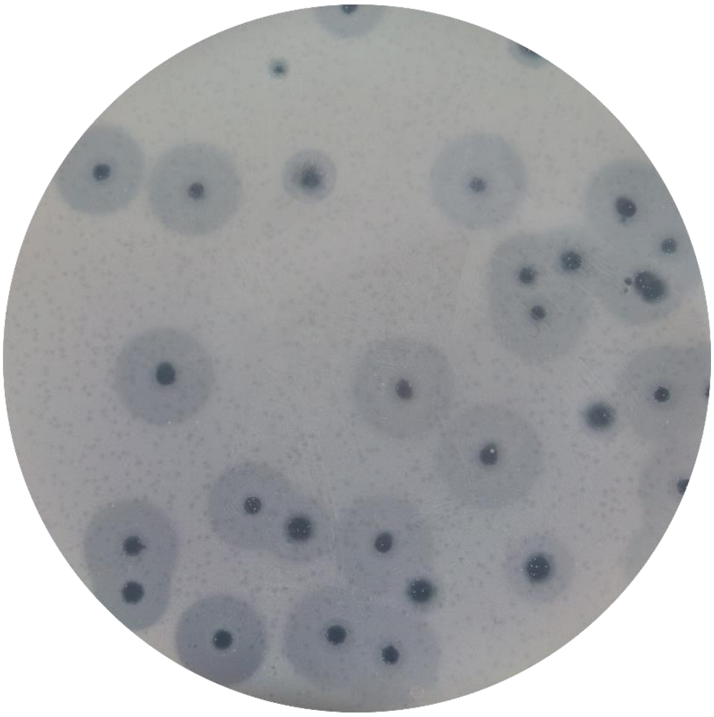

Supplementary Figure S1. Plaque assay of *Kp* 7984 with phage TUN1 assembled in *E. coli* NEBstable cells. After being assembled in a non-replicative host, phage TUN1 causes plaques on *Kp* 7984.
